# Aqueous Extract of *Limnospira platensis* Provides Protection Against Microcystin-Induced Oxidative Stress in Hydroponic Culture of Radish (*Raphanus sativus*)

**DOI:** 10.3390/jox15060182

**Published:** 2025-11-01

**Authors:** Mohammed Haida, Badr Ezzyky, Zineb Hakkoum, Richard Mugani, Yasser Essadki, Fatima El Khalloufi, Abdelmajid Haddioui, Mohamed Loukid, Brahim Oudra, Noureddine Bouaïcha

**Affiliations:** 1 Laboratory of Aquatic Sciences, Microbial Biotechnology and Sustainability of Natural Resources, Department of Biology, Faculty of Sciences Semlalia, Cadi Ayyad University (UCA), Marrakesh 40000, Morocco; mohammed.haida11@gmail.com (M.H.); zineb.hakkoum@gmail.com (Z.H.); richardmugani@gmail.com (R.M.); yasser.essadki@ced.uca.ma (Y.E.); oudra@uca.ac.ma (B.O.); 2 Laboratory of Agro-Industrial and Medical Biotechnologies, Faculty of Sciences and Technics, Sultan Moulay Slimane University, Beni Mellal 23000, Morocco; badrezzyky75@gmail.com (B.E.);; 3 National Institute of Public Health, Ministry of Health, Avenue Ruvubu, Bujumbura 6807, Burundi; 4 Natural Resources Engineering and Environmental Impacts Team, Multidisciplinary Research and Innovation Laboratory, Polydisciplinary Faculty of Khouribga, Sultan Moulay Slimane University of Beni Mellal, Khouribga 25000, Morocco; f.elkhalloufi@usms.ma; 5 Laboratory of Pharmacology, Neurobiology, Anthropobiology and Environment, Department of Biology, Faculty of Sciences Semlalia, Cadi Ayyad University (UCA), Marrakesh 40000, Morocco; loukid@uca.ac.ma; 6 Laboratoire Écologie, Société et Évolution, UMR 8079, Université Paris-Saclay, CNRS, AgroParisTech, 91190 Gif-sur-Yvette, France

**Keywords:** cyanobacteria, microcystins (MCs), *Microcystis aeruginosa*, *Limnospira platensis*, biostimulation, bioprotection, *Raphanus sativus*, oxidative stress

## Abstract

The eutrophication of aquatic ecosystems often triggers the excessive growth of cyanobacteria, many of which release toxic metabolites such as microcystins (MCs). When irrigation water is contaminated by these compounds, adverse consequences may arise for plants as well as for animal and human health. In contrast, certain non-toxic cyanobacterial species like *Limnospira platensis* are increasingly regarded as valuable tools for sustainable agriculture, given their ability to enhance plant nutrition, growth, yield, and stress tolerance while also mitigating the detrimental impacts of MCs. The present work aimed to investigate the potential of *L. platensis* extract to enhance growth, physiological responses, and tolerance of radish (*Raphanus sativus*) plants stressed with *Microcystis aeruginosa* extract containing microcystins. Experiments were conducted in a hydroponic system under controlled environmental conditions, where radish seedlings were cultivated in perlite and exposed for 45 days to *M. aeruginosa* extract (10 and 40 µg/L of MCs) and *L. platensis* extract (0.1 and 1 g/L), applied either separately or in combination. The results showed that the application of *L. platensis* extract, especially at 1 g/L in combination with 40 µg/L of MCs, decreased the bioaccumulation of MCs from 8.81 to 5.35 µg/kg FW in the leaves and from 14.64 to 10.15 µg/kg FW in the taproots. In addition, it significantly stimulated radish growth and improved several biochemical parameters. In contrast, exposure to MCs at 10 and 40 µg/L negatively affected growth, chlorophyll pigments and protein contents while promoting the accumulation of malondialdehyde (MDA), polyphenols and sugars. The activities of peroxidase (POD), superoxide dismutase (SOD), and catalase (CAT) were also increased under MCs stress, suggesting activation of the antioxidant defense system in response to oxidative damage. Combinations of MCs with *L. platensis* extract, especially at 1 g/L, improved antioxidant enzyme activities by significantly reducing MDA levels, biometric parameters, chlorophyll pigment, and protein and sugar contents. These results indicate that the application of *L. platensis* extract as a biostimulant can improve radish development, growth, and tolerance to MC-induced stress.

## 1. Introduction

The issue of water availability and quality is becoming increasingly critical in semi-arid Mediterranean countries, where the incidence of cyanobacterial harmful algal blooms (cyano-HABs) has been exacerbated by the synergistic effects of eutrophication and climate change [1,2]. The majority of cyanobacteria involved in bloom formation are recognized as potential cyanotoxin producers. Among the most frequently encountered cyanotoxins are microcystins (MCs), part of the hepatotoxin group, generally produced by *Microcystis aeruginosa* [3]. To date, 279 structural variants have been identified, with microcystin-LR (MC-LR) reported as the most prevalent [4]. MCs act as strong inhibitors of protein phosphatases 1 and 2A, enzymes that play key roles in numerous physiological and molecular processes in both animals and higher plants [5]. Globally, the highest reported concentration of MCs (2,900,000 µg/L) was detected in cyanobacterial scums from a *Microcystis* sp. bloom in Pinto Lake, California [6]. In Morocco, specifically in the Lalla Takerskoust reservoir, concentrations of the extracellular and intracellular fractions of MCs in water can reach 281 µg/L and 10,257 µg/g, respectively [7]. This untreated water, containing high MC concentrations, is primarily used for irrigation without any regulation or monitoring. Therefore, this practice is likely to cause phytotoxic effects, including reduced seed germination, impaired growth and development, and decreased photosynthetic performance [8,9]. Such toxins are capable of altering plant metabolic processes, leading to increased lipid peroxidation, decreased protein levels, and triggering oxidative stress [9]. However, reactive oxygen species (ROS) are inevitably produced under stress conditions and can cause severe oxidative damage to proteins, lipids, and photosynthetic pigments. To counteract this, plants rely on an efficient antioxidant defense system, including enzymes such as superoxide dismutase (SOD), catalase (CAT), and peroxidase (POD). SOD converts superoxide radicals into hydrogen peroxide, while CAT and POD subsequently detoxify hydrogen peroxide into water and oxygen, thereby maintaining cellular homeostasis and protecting the photosynthetic machinery [9,10].

Improving plant growth and nutritional quality in MC-contaminated environments requires effective management of irrigation water. Physical and chemical techniques, such as flocculation [11], ultrasonic treatment [12], and copper sulfate application [13], have been developed to control cyanobacterial blooms. However, these methods often present limitations, including high energy costs, limited long-term effectiveness, and potential environmental side effects. To address these issues, biological alternatives based on the use of microorganisms have attracted growing attention in recent years. Most research on the biodegradation of MCs has focused on microorganisms isolated from water sources that have experienced algal blooms [14]. In this context of biological solutions, the use of biostimulants derived from non-MC-producing cyanobacterial species, such as *L. platensis*, offers a sustainable approach to safely improve crop production [15]. Biostimulants have been shown to enhance rooting, nutrient uptake, seedling establishment, and plant tolerance to a wide range of biotic and abiotic stresses [16,17]. These beneficial effects are largely attributed to their content in growth regulators, including phytohormones, vitamins, amino acids, and polysaccharides, which are essential for plant development [18]. In this context, the present study investigates the potential of an aqueous extract of *L. platensis* as a biotechnological tool to stimulate growth and productivity while mitigating MC-induced stress in hydroponically grown radish (*Raphanus sativus*) under controlled conditions.

## 2. Materials and Methods

### 2.1. Preparation of M. aeruginosa and L. platensis Extracts

To obtain cyanobacterial extracts, 10 g of dried *L. platensis* biomass previously collected from Lake Chad was suspended in 100 mL of distilled water and agitated continuously at 60 °C for 15 min. The extract was filtered through a 0.22 µm Millipore membrane and stored at 4 °C. This stock solution, corresponding to 100 g dry weight (DW)/L, was subsequently diluted to prepare the working concentrations of 0.1 and 1 g (DW)/L. Samples of *M. aeruginosa* bloom were collected in October 2010 from the Lalla Takerkoust reservoir, Marrakech, Morocco (31°36′ N, 8°20′ W, 664 m), and freeze-dried. For extraction, 250 mg of freeze-dried material was ground in a mortar with 50 mL of distilled water and subjected to cold sonication (42 kHz, 5 min) to release intracellular microcystins (MCs). After centrifugation at 10,000× *g* for 15 min, the supernatant was collected, while the pellet was re-extracted twice under identical conditions. All supernatants were pooled and stored at −20 °C until analysis. The total MC content in the aqueous extract was determined using the serine/threonine protein phosphatase type 2A inhibition assay as described by Bouaïcha et al. [19]. In brief, the assay is based on the dephosphorylation of para-nitrophenylphosphate (p-NPP), generating para-nitrophenol, a colored compound. Enzymatic activity is quantified by monitoring the formation of this product at 405 nm, thereby enabling evaluation of the inhibitory effect of MCs on PP2A-mediated p-NPP dephosphorylation. Using this method, the concentration of MCs in the *M. aeruginosa* bloom extract was determined as 11.5 mg MC-LR equivalents per g DW. Complementary qualitative analysis performed with high-performance liquid chromatography (HPLC) coupled to mass spectrometry showed that MC-LR was the dominant variant, accounting for 98% of the total MCs [20]. Two MC concentrations (10 and 40 µg/L) were freshly prepared for each application in plant irrigation during the treatment period.

### 2.2. Biochemical Characterization of M. aeruginosa, L. platensis, and Raphanus sativus Tissue Samples

#### 2.2.1. Total Soluble Sugars

To determine soluble sugar concentrations in *M. aeruginosa*, *L. platensis*, and *Raphanus sativus* tissues, 100 mg of sample material was extracted with 4 mL of 80% ethanol and centrifuged at 5000× *g* for 10 min. From the supernatant, 1 mL was combined with 1 mL of 5% phenol and 5 mL of concentrated sulfuric acid. After incubation for 5 min, the absorbance of the mixture was recorded at 485 nm. Glucose was used as the calibration standard [21], and soluble sugar content was expressed as mg glucose equivalents per g DW (*M. aeruginosa* and *L. platensis*) or per g FW (radish leaves, roots, and taproots).

#### 2.2.2. Total Protein Contents

An aliquot of 500 mg of dried *M. aeruginosa*, *L. platensis*, and *Raphanus sativus* tissue samples was ground in liquid nitrogen in a cold mortar. The resulting powder was homogenized in 5 mL of buffer composed of 50 mM potassium phosphate (pH 7.0), 5% (*w*/*v*) polyvinylpolypyrrolidone (PVPP), and 0.1 mM EDTA. The homogenates were centrifuged at 12,500× *g* for 20 min at 4 °C, and the supernatants were collected for subsequent analyses of protein content and antioxidant enzyme activities following the method of Savicka et al. [22].

For proteins quantification, 100 µL of each extract was combined with 2 mL of Bradford reagent. The mixtures were incubated at room temperature for 5 min, after which absorbance was measured at 595 nm. Protein concentrations were calculated against a standard curve prepared with bovine serum albumin (BSA) [23]. Each measurement was performed in triplicate, and results were expressed as mg BSA equivalent per g DW (*M. aeruginosa*, *L. platensis*) or per g FW (radish leaves, roots, and taproots). The analysis of antioxidant enzyme activities on fresh leaves and roots samples is described in Section 2.6.4.

#### 2.2.3. Total Polyphenols

The total polyphenol content of dried *M. aeruginosa*, *L. platensis*, and *Raphanus sativus* tissues was quantified using the colorimetric method described by Singleton et al. [24]. For the assay, 0.2 mL of each extract was mixed with 2.4 mL of distilled water and 0.4 mL of Folin–Ciocalteu reagent. After vortexing, the mixture was left to react for 3 min, followed by the addition of 1 mL of 20% (*w*/*v*) sodium carbonate (Na_2_CO_3_). The samples were vortexed again and incubated for 1 h at room temperature in the dark. Absorbance was subsequently measured at 765 nm. Quantification was performed using a calibration curve prepared with gallic acid (0–200 µg/mL). All measurements were carried out in triplicate, and results were expressed as µg gallic acid equivalents per g DW (*M. aeruginosa* and *L. platensis*) or per g FW (radish leaves, roots, and taproots).

#### 2.2.4. Flavonoids

The total flavonoid contents in dried *M. aeruginosa* and *L. platensis* extract samples were determined using a colorimetric assay with aluminum trichloride (AlCl_3_) according to Kim et al. [25]. For each assay, 500 µL of aqueous extract were mixed with 1.5 mL of distilled water and 150 µL of 5% sodium nitrite (NaNO_2_) solution. After a 5-min incubation, 150 µL of 10% AlCl_3_ (prepared in methanol (MeOH)) was added. After 11 min of incubation, 500 µL of 1 M NaOH was introduced, and the mixture was vortexed. Absorbance was recorded immediately at 510 nm. Quantification was carried out using a calibration curve constructed with catechin standards (0–200 µg/mL). Each measurement was performed in triplicate, and results were expressed as µg catechin equivalents per g DW.

#### 2.2.5. Auxins

Auxin levels in dried *M. aeruginosa* and *L. platensis* extracts were quantified using the Salkowski colorimetric method following Gang et al. [26]. For the assay, 3 mL of aqueous extract was mixed with 2 mL of Salkowski reagent, vortexed, and kept in the dark at room temperature for 30 min. The absorbance of the resulting solution was recorded at 536 nm. Concentrations were calculated from a calibration curve prepared with pure indole-3-acetic acid (IAA). Each determination was carried out in triplicate, and results were expressed as mg IAA equivalents per g DW.

### 2.3. Culture of Radish Plants and Treatment with Different Extracts

Certified seeds of *Raphanus sativus* (national variety) were obtained from BADRA S.A.R.L, Casablanca, Morocco. The seeds were rinsed with distilled water, and germinated individually in plastic cells filled with peat. After 10 days, when seedlings had developed two to three leaves, they were transplanted into 0.5 L plastic bags containing perlite, a substrate commonly used in hydroponics. Plants were cultivated for 45 days in a growth chamber under controlled conditions (20 ± 1 °C, light intensity of 200 µmol m^−2^ s^−1^, 13/11 h light/dark cycle, relative humidity 70–80%). The 54 pots were assigned to nine experimental treatments:

- C (control): irrigation with alternating water and nutrient solution,
- C1: irrigation with water containing 10 µg/L of MCs,
- C2: irrigation with water containing 40 µg/L of MCs,
- B1: irrigation with water containing 0.1 g/L *L. platensis* extract,
- B2: irrigation with water containing 1 g/L *L. platensis* extract,
- C1 + B1: irrigation with water containing 10 µg/L of MCs + 0.1 g/L *L. platensis* extract,
- C1 + B2: irrigation with water containing 10 µg/L of MCs + 1 g/L *L. platensis* extract,
- C2 + B1: irrigation with water containing 40 µg/L of MCs + 0.1 g/L *L. platensis* extract,
- C2 + B2: irrigation with water containing 40 µg/L of MCs + 1 g/L *L. platensis* extract.

To minimize positional effects, pots were randomly rearranged every 7 days. The experiment was conducted with six replicates per treatment (one plant per pot). Plants were irrigated with 75 mL of Hoagland’s nutrient solution [27] alternated with water containing *L. platensis* and/or MC extracts at two-day intervals. After 45 days, biomass was harvested to assess growth traits. Plant tissues (leaves, roots, and taproots) were separated, stored at −20 °C for biochemical analyses, and selected samples (leaves and taproots) were reserved for MCs detection.

### 2.4. Determination of MCs in Radish Tissues

To evaluate the bioaccumulation of MCs in radish tissues, 1 g of leaves or 2 g of taproots was ground in liquid nitrogen and freeze-dried. Samples were homogenized in 5 mL (leaves) or 10 mL (taproots) of 75% (*v*/*v*) aqueous methanol. Following centrifugation at 4000× *g* for 10 min at 4 °C, the supernatants were collected, and the residues were re-extracted twice under the same conditions. The combined supernatants were then purified using C18 solid-phase extraction (SPE). After, the cartridges were conditioned with 5 mL methanol followed by 5 mL deionized water. Elution of MCs was performed with 5 mL methanol/formic acid (95:5, *v*/*v*). The eluates were evaporated to dryness in a rotary evaporator, and the residues were resuspended in 1 mL deionized water before storage at −80 °C until ELISA analysis. MC concentrations in radish leaves and taproots were expressed as µg MC-LR equivalents per kg FW [28].

### 2.5. Determination of Biometric Parameters and Yield Parameters of Radish Plants

After 45 days of exposure with the different treatments, plant biomass was harvested (at fruit stage). The taproots and aerial sections were separated to determine various growth metrics. To evaluate the effect of different treatments on radish plant growth, total root length, aerial part height and yield parameters (taproots) were measured using a ruler and caliper. The results were expressed in centimeters (cm). Furthermore, the number of leaves per plant was counted for all treatments. For leaf area determination, leaves were sliced, put on a white sheet with a scale, scanned using a digital scanner, and the area was determined using the Mesurum software version 3.4.4.0 [29]. For biomass determination, aerial parts, roots, and taproots were carefully rinsed with water to eliminate perlite residues, blotted dry with absorbent paper, and weighed using a precision balance, and the results were expressed in grams (g).

### 2.6. Determination of Biochemical Parameters in Radish Tissues

#### 2.6.1. Pigment Content in Leaves

After 45 days of treatment, radish plants were harvested and their leaves were collected for the determination of photosynthetic pigments, including chlorophyll a (Chl a), chlorophyll b (Chl b), total chlorophyll, and carotenoids. For pigment extraction, 0.5 g of fresh leaf tissue was homogenized in 5 mL of 95.5% acetone. The samples were incubated in darkness for 48 h, after which absorbance was recorded at 470, 644, and 662 nm, following the procedure described in [30]. Pigment concentrations (mg/g FW) were then calculated using the standard equations.

Chl a = 9.784 (OD 662) − 0.99 (OD 644)

Chl b = 21.42 (OD 644) − 4.65 (OD 662)

Total chlorophyll = Chl a + Chl b

Carotenoids = 1000 (OD470) − 1.90 Chl a − 63.14 Chl b/214

#### 2.6.2. Vitamin C Content in Taproots

After 45 days of treatment, radish plants were harvested and the taproots were used for the determination of ascorbic acid content through a titrimetric assay. 0.5 g of fresh taproot tissue was ground in a mortar and extracted with 3 mL of 2% HCl, followed by a 10 min incubation. The homogenate was centrifuged at 5000× *g* for 10 min at 4 °C, and the supernatant was diluted with 3 mL of distilled water. A few drops of 0.5% starch solution were added as an indicator, and the mixture was titrated with 0.01 N iodine solution until the appearance of the characteristic dark blue-black color, which marked the endpoint [31]. The concentration of vitamin C was calculated and expressed as mg ascorbic acid equivalent per 100 g FW.

#### 2.6.3. Inorganic Ions in Taproots

The collected taproots were analyzed to determine the concentrations of inorganic ions, including calcium (Ca), magnesium (Mg), zinc (Zn), potassium (K), and iron (Fe). For this purpose, 0.25 g of freeze-dried material was subjected to calcination in a muffle furnace at 550 °C for 6 h. The resulting ash was solubilized in 3 mL of 6 N HCl, then evaporated at 250 °C under a fume hood. The residue was dissolved in 3 mL of preheated distilled water (95 °C) and filtered through 0.45 μm Whatman paper. Each sample was subsequently brought to a final volume of 50 mL with hot distilled water (95 °C). Mineral concentrations (Ca, Mg, Zn, K, and Fe) were quantified using inductively coupled plasma (ICP) analysis according to [32], while total phosphorus content was determined following the method of Olsen and Sommers [33]. Results were expressed as mg/100 g DW.

#### 2.6.4. Antioxidant Enzyme Activities in Radish Tissues

After 45 days of exposure to the different treatments, radish plants were collected and separated into leaves and roots to assess the activities of antioxidant enzymes: peroxidase (POD), superoxide dismutase (SOD), and catalase (CAT). POD activity was determined using a reaction mixture consisting of 100 µL of leaf or root extracts (prepared in 10 mM phosphate buffer, pH 6), 0.25% guaiacol, and 20 mM H_2_O_2_. The oxidation of guaiacol was monitored at 470 nm following the method of [34], and the activity was expressed as Unit/mg protein. For SOD activity was assayed according to [35] with slight modifications. The reaction mixture contained 200 µL of extracts prepared in 100 mM phosphate buffer (pH 7.8), 0.75 mM nitroblue tetrazolium, 0.1 mM riboflavin, and 55 mM methionine. After incubation at 25 °C for 20 min, the absorbance was measured at 560 nm, and results were expressed as Unit/mg protein. CAT activity was quantified using extracts prepared in 50 mM phosphate buffer (pH 7.0). The reaction mixture (200 µL of extract, 15 mM H_2_O_2_, and 2.6 mL of buffer) was used to measure the degradation of hydrogen peroxide at 240 nm, as described by [36]. The activity was expressed as µmol H_2_O_2_ decomposed/min/mg protein.

#### 2.6.5. Determination of Malondialdehyde (MDA) in Radish Tissues

The level of malondialdehyde (MDA), an indicator of lipid peroxidation, was assessed using the thiobarbituric acid (TBA) assay as outlined in [22]. Fresh leaf and root tissues (0.2 g) were homogenized in 2 mL of 0.1% trichloroacetic acid (TCA), and the homogenate was centrifuged at 14,000× *g* for 15 min. To 1 mL of the obtained supernatant, 2.5 mL of 20% TCA containing 0.5% TBA was added. The mixture was heated at 95 °C for 30 min, then immediately cooled in an ice bath. After a second centrifugation at 10,000× *g* for 30 min, absorbance of the clear supernatant was recorded at 532 and 600 nm. MDA concentration was calculated and expressed as µmol/g fresh weight (FW).

### 2.7. Statistical Analysis

All data were tested for normality (Shapiro–Wilk test) and homogeneity of variances (Levene’s test) prior to analysis. Measurements were expressed as mean ± standard error (SE). Statistical differences among treatments were assessed using one-way ANOVA. When significant differences were detected, multiple comparisons were performed using Tukey’s HSD post hoc test. Groups marked with different letters were considered significantly different at *p* < 0.05. All analyses were conducted using IBM SPSS Statistics software, version 26.0 (IBM Corp., Armonk, NY, USA).

## 3. Results

### 3.1. Characterization of M. aeruginosa and L. platensis Extracts

Table 1 presents the biochemical properties of the aqueous extracts of *L. platensis* and *M. aeruginosa*. The extract of *L. platensis* contains high contents of soluble sugars (15.23 mg/g DW), proteins (39.67 mg/g DW) and auxins (44.69 mg/g DW), as well as low amounts of total phenols (0.2 µg Gallic acid/g DW) and flavonoids (0.3 µg Catechin/g DW). In contrast, the extract of *M. aeruginosa* contains higher soluble sugars (25.62 mg/g DW) and a little less proteins (30.23 mg/g DW), while total flavonoids, auxins, and phenols are not detected in this extract. These findings highlight clear differences in the biochemical profiles of the two cyanobacterial extracts, with some bioactive compounds—including auxins, phenols, and flavonoids—being detected exclusively in *L. platensis* extract (Table 1).

### 3.2. Bioaccumulation of MCs in Radish Tissues

Table 2 presents the levels of MCs accumulated in radish leaves and taproots, as determined by the ELISA assay. After 45 days of exposure to water contaminated with MCs (10 and 40 µg/L) showed an increase in the bioaccumulation of MCs in both organs compared to the control. The concentrations ranged from 10.13 to 14.64 µg equiv. MC-LR/kg FW in taproots and from 7.33 to 8.81 µg equiv. MC-LR/kg FW in leaves (Table 2). However, when radish plants were simultaneously exposed to both cyanobacterial extracts, treatment with *L. platensis* at 0.1 or 1 g/L resulted in a modest, though statistically non-significant, reduction in MC accumulation in both leaves and taproots.

### 3.3. Effects of L. platensis Extract on Radish Growth Under MC-Induced Stress

Table 3 summarizes the impact of the different treatments on radish growth parameters after 45 days of exposure to MC-induced stress. Application of MCs alone, at either tested concentration, did not significantly affect leaf number (LN), leaf area (LA), leaf fresh weight (LFW), or root fresh weight (RFW) compared with the control. By contrast, supplementation with *L. platensis* extract (0.1 and 1 g/L) led to a significant increase (*p* < 0.001) in LN relative to the control. Moreover, co-application of 10 µg/L MCs + 1 g/L *L. platensis* or 40 µg/L MCs + 1 g/L *L. platensis* significantly enhanced LA compared with plants treated with MCs alone. A notable increase in LFW was observed in plants receiving 40 µg/L MCs combined with 1 g/L *L. platensis* compared with the control. Similarly, RFW was significantly higher (*p* < 0.001) in plants treated with 1 g/L of *L. platensis,* 10 µg/L MCs + 1 g/L of *L. platensis* and 40 µg/L MCs + 1 g/L *L. platensis*, compared with the control (Table 3). Treatment of radish plants with either MCs or *L. platensis* extracts alone did not significantly affect taproot length (TL) (*p* = 0.276) or diameter (TD) (*p* < 0.001) (Table 3). A significant increase in taproot fresh weight (TFW) and dry weight (TDW) was observed only with the higher concentration of *L. platensis* extract (1 g/L), whereas exposure to 40 µg/L MCs led to a significant reduction (*p* < 0.001) in taproot dry weight (TDW) compared with control. In contrast, combined application of MCs and *L. platensis* extracts, at the tested concentrations, produced no significant changes in taproot yield (Table 3).

### 3.4. Effect of L. platensis Extract on the Biochemical Parameters of Radish-Stressed Tissues by MCs

#### 3.4.1. Photosynthetic Pigment Content

**Figure 1:**
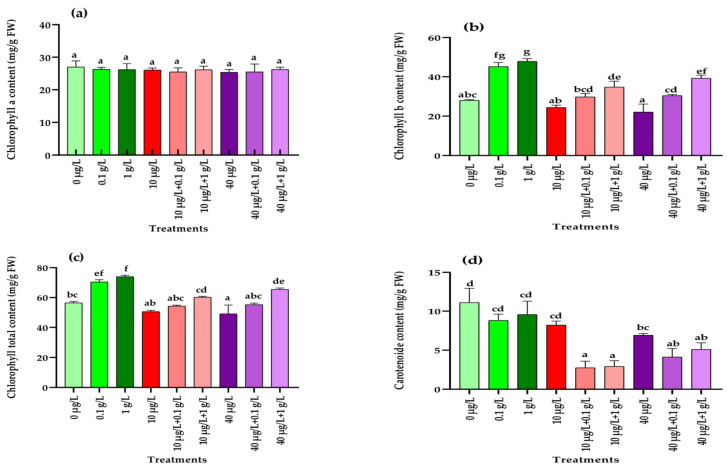
Photosynthetic pigment contents (mg/g FW) in radish leaves after 45 days of treatment with 0 µg/L (water and nutrient solution), *L. platensis* extract (0.1 and 1 g/L), MCs (10 and 40 µg/L), and their combinations. (**a**) Chlorophyll a, (**b**) chlorophyll b, (**c**) total chlorophyll, and (**d**) carotenoids. Bars represent mean values ± standard deviation (SD). Different letters indicate significant differences (*p* < 0.05, Tukey’s HSD test).

Figure 1 illustrates the effects of the different treatments on photosynthetic pigment contents in radish plants exposed to MC-induced stress. No significant differences (*p* = 0.872) in chlorophyll a were detected among treatments with MCs, *L. platensis* extracts, or their combinations compared with the control (Figure 1a). In contrast, chlorophyll b significantly increased (*p* < 0.001) in response to both concentrations of *L. platensis* extract (0.1 and 1 g/L), whereas MCs alone (10 and 40 µg/L) had no effect (Figure 1b). Co-treatments combining MCs with 1 g/L of *L. platensis* also enhanced chlorophyll b, although to a lesser extent than the extract applied alone. A similar pattern was observed for total chlorophyll content (Figure 1c). For carotenoids, neither *L. platensis* extract nor 10 µg/L MCs significantly altered (*p* < 0.001) levels, while the highest MC concentration (40 µg/L) and all co-treatments caused a significant reduction compared with the control (Figure 1d).

#### 3.4.2. Total Soluble Sugar Content

**Figure 2:**
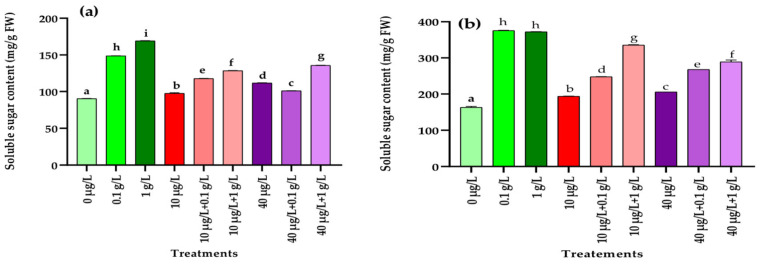
Total sugar content (mg/g FW) in leaves (**a**) and roots (**b**) of the radish leaves after 45 days of treatment with 0 µg/L (water and nutrient solution), *L. platensis* extract (0.1 and 1 g/L), MCs (10 and 40 µg/L), and their combinations. Bars represent mean values ± SD. Different letters indicate significant differences (*p* < 0.05, Tukey’s HSD test).

The impact of MCs and *L. platensis* extracts, applied either individually or in combination, on soluble sugar levels in radish leaves and roots is illustrated in Figure 2. In the leaves, all treatments resulted in a significant increase (*p* < 0.001) in soluble sugar content compared with the control, with the strongest induction observed in plants treated with both concentrations of *L. platensis* extract alone (Figure 2a). The combination of MC and *L. platensis* extract showed also a significant increase (*p* < 0.001) of total sugar contents compared to the control and to the treatment with MCs extract alone, but remain significantly low than those induced by the *L. platensis* extract alone. The same trend of results was also observed for the roots (Figure 2b).

#### 3.4.3. Total Proteins Content

**Figure 3:**
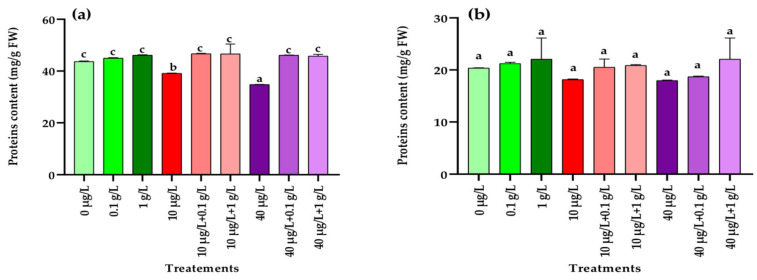
Protein contents (mg/g FW) in leaves (**a**) and roots (**b**) of the radish leaves after 45 days of treatment with 0 µg/L (water and nutrient solution), *L. platensis* extract (0.1 and 1 g/L), MCs (10 and 40 µg/L), and their combinations. Bars represent mean values ± SD. Different letters indicate significant differences (*p* < 0.05, Tukey’s HSD test).

Total proteins content obtained from radish leaves and roots treated with the different extracts alone or in combination are presented in Figure 3. In the leaves, exposure to MCs at both concentrations (10 and 40 µg/L) caused a dose-dependent decline in total protein content compared with the control (Figure 3a). However, co-application of *L. platensis* extract with MCs led to a significant recovery (*p* < 0.001) of protein levels, restoring them close to the control values (Figure 3a). In contrast, no significant (*p* = 0.122) variations were observed in root protein content across the different treatments compared with the control (Figure 3b).

### 3.5. Effect of L. platensis Extract on Stress Markers of Radish-Stressed by MCs

#### 3.5.1. Total Polyphenols Content

**Figure 4:**
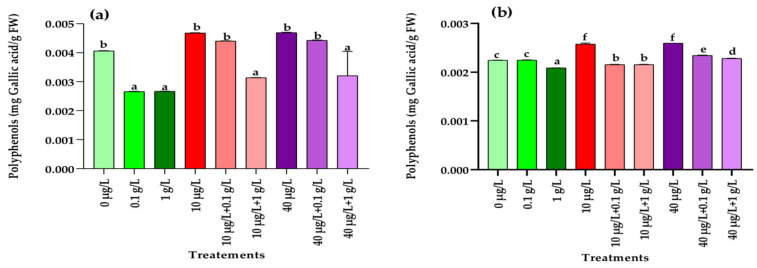
Polyphenol contents (mg Gallic acid/g FW) in leaves (**a**) and roots (**b**) of the radish leaves after 45 days of treatment with 0 µg/L (water and nutrient solution), *L. platensis* extract (0.1 and 1 g/L), MCs (10 and 40 µg/L), and their combinations. Bars represent mean values ± SD. Different letters indicate significant differences (*p* < 0.05, Tukey’s HSD test).

Exposure to MCs at both concentrations (10 and 40 µg/L) resulted in a significant increase (*p* < 0.001) in polyphenol levels in radish leaves and roots compared with the control (Figure 4a,b). In contrast, treatment with *L. platensis* extract at both concentrations (0.1 and 1 g/L) caused a significant reduction (*p* < 0.001) in leaf polyphenols, while in roots this effect was observed only at the higher concentration (1 g/L) (Figure 4b). Under combined treatments, a significant decrease (*p* < 0.001) in leaf polyphenols compared to the control was detected only when MCs were applied together with the highest concentration (1 g/L) of *L. platensis* extract (Figure 4a). In roots, co-treatment with 10 µg/L of MCs and both concentrations of *L. platensis* extract significantly reduced (*p* < 0.001) polyphenol content, whereas the combination of 40 µg/L of MCs with both *L. platensis* concentrations significantly increased (*p* < 0.001) it compared with the control (Figure 4b).

#### 3.5.2. Determination of Malondialdehyde (MDA)

**Figure 5:**
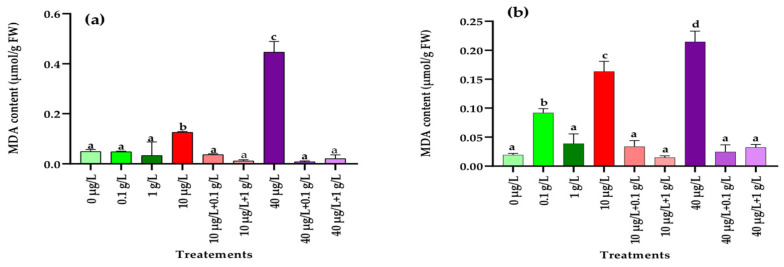
Malondialdehyde content (MDA) (µmol/g FW) in leaves (**a**) and roots (**b**) of the radish leaves after 45 days of treatment with 0 µg/L (water and nutrient solution), *L. platensis* extract (0.1 and 1 g/L), MCs (10 and 40 µg/L), and their combinations. Bars represent mean values ± SD. Different letters indicate significant differences (*p* < 0.05, Tukey’s HSD test).

As shown in Figure 5, a significant (*p* < 0.001) rise in malondialdehyde (MDA) levels compared to the control was detected only in radish roots treated with the lower concentration (0.1 g/L) of *L. platensis* extract (Figure 5b). In contrast, both MC concentrations (10 and 40 µg/L) caused a marked increase in MDA content in leaves and roots (Figure 5a,b). However, when plants were co-exposed to MCs and *L. platensis* extracts, the presence of *L. platensis*, at both concentrations, restored MDA levels in both tissues to values comparable to the control (Figure 5a,b).

#### 3.5.3. Peroxidase (POD) Activity

**Figure 6:**
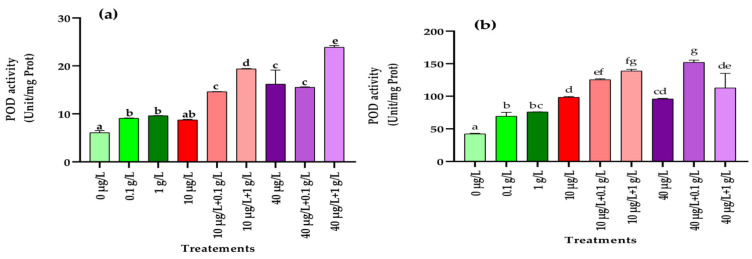
Peroxidase (POD) activity (Unit/mg Prot.) in leaves (**a**) and roots (**b**) of the radish leaves after 45 days of treatment with 0 µg/L (water and nutrient solution), *L. platensis* extract (0.1 and 1 g/L), MCs (10 and 40 µg/L), and their combinations. Bars represent mean values ± SD. Different letters indicate significant differences (*p* < 0.05, Tukey’s HSD test).

Application of *L. platensis* extract at both tested concentrations (0.1 and 1 g/L) significantly enhanced POD activity in radish leaves and roots compared with the control. Similarly, exposure to the highest MC concentration (40 µg/L) also led to a marked increase in this enzymatic activity in both tissues, whereas the lower dose (10 µg/L) significantly stimulated POD activity only in roots (Figure 6a,b). Interestingly, co-application of *L. platensis* and MCs extracts further amplified POD activity in both leaves and roots, exceeding not only the control values but also those recorded under single treatments (Figure 6a,b).

#### 3.5.4. Superoxide Dismutase (SOD) Activity

**Figure 7:**
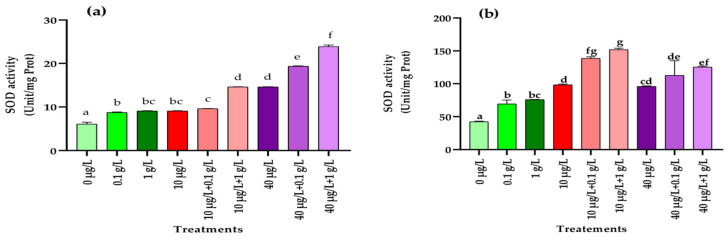
Superoxidase dismutase (SOD) (Unit/mg Prot.) in leaves (**a**) and roots (**b**) of the radish leaves after 45 days of treatment with 0 µg/L (water and nutrient solution), *L. platensis* extract (0.1 and 1 g/L), MCs (10 and 40 µg/L), and their combinations. Bars represent mean values ± SD. Different letters indicate significant differences (*p* < 0.05, Tukey’s HSD test).

As shown in Figure 7, exposure to both *L. platensis* and MC extracts significantly enhanced (*p* < 0.001) superoxide dismutase (SOD) activity in radish leaves and roots compared with the control. The increase was even more pronounced under combined treatments, where co-application of the two extracts resulted in higher SOD activity than in the control or plants treated with *L. platensis* extract alone.

#### 3.5.5. Catalase (CAT) Activity

**Figure 8:**
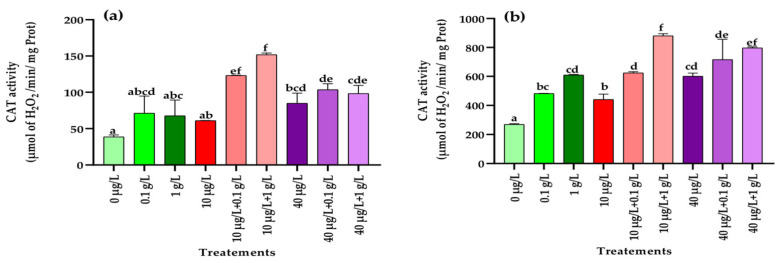
Catalase (CAT) (µmol of H_2_O_2_/min/mg Prot.) in leaves (**a**) and roots (**b**) of the radish leaves after 45 days of treatment with 0 µg/L (water and nutrient solution), *L. platensis* extract (0.1 and 1 g/L), MCs (10 and 40 µg/L), and their combinations. Bars represent mean values ± SD. Different letters indicate significant differences (*p* < 0.05, Tukey’s HSD test).

As illustrated in Figure 8a, a significant increase (*p* < 0.001) in CAT activity in leaves was detected only at the highest concentration of MC extract (40 µg/L) compared with the control. In contrast, in roots, CAT activity was significantly enhanced (*p* < 0.001) when plants were exposed to both *L. platensis* and MC extracts (Figure 8b). Co-application of the lowest MC concentration (10 µg/L) with either 0.1 or 1 g/L of *L. platensis* extract strongly stimulated CAT activity in both leaves and roots, surpassing the control and the effects of each extract applied separately. For the highest MC concentration (40 µg/L), co-treatment with *L. platensis* extracts also resulted in higher CAT activity compared to the control, but the strongest induction compared with MC extract alone was observed only in the roots with the highest *L. platensis* concentration (1 g/L) (Figure 8b).

### 3.6. Effects of L. platensis Extract on Biochemical Parameters of Radish Taproots Under MC-Induced Stress

Regarding the biochemical parameters of radish taproots, the results showed that soluble sugar, proteins, and polyphenols levels exhibited no significant effects in all applied treatments compared to the control. However, the vitamin C content was decreased notably under MCs extract (10 and 40 µg/L) exposure in comparison to the control. Controversy, it exhibited a significant increase with *L. platensis* extracts (0.1 and 1 g/L) compared to the control. A significant increase (*p* < 0.001) of the vitamin C level was also recorded for treatments with 0.1 g/L, 1 g/L of the *L. platensis* extract, and 10 µg/L MCs + 0.1 of the *L. platensis* extract compared to the control (Table 4). In contrast, when the radish plant was co-treated with the high concentration of MC extract (40 µg/L) and both concentrations (0.1 and 1 g/L) of *L. platensis* extract, a significant increase (*p* < 0.001) in vitamin C level was also recorded compared to the treatment with 40 µg/L MC (Table 4).

### 3.7. Effects of L. platensis Extract on Nutrient Content of Radish Taproots Under MC-Induced Stress

The results presented in Table 5 indicate that the taproots of plants treated with 40 µg/L of MC extract or with the different combinations between MC and *L. platensis* showed a significant reduction (*p* < 0.001) in potassium (K) content compared to the control. In contrast, treatment with the biostimulant concentrations (0.1 and 1 g/L) of *L. platensis* extract resulted in a significant increase (*p* < 0.001) in K levels compared to treatment with MC alone. Regarding magnesium (Mg), both concentrations of *L. platensis* extract induced a significant decrease compared to the control, a reduction that was also evident under treatment with the high concentration (40 µg/L) of MC extract. Similarly, co-treatments with both 10 and 40 µg/L MC extracts and 1 g/L *L. platensis* extract led to a significant decrease (*p* < 0.001) in Mg content compared to the control. Treatment of radish plants with both concentrations of MC extract resulted in a significant reduction (*p* < 0.001) of zinc (Zn) content in taproots compared to the control. Conversely, application of both concentrations of *L. platensis* extract significantly increased (*p* < 0.001) in Zn levels. In co-treatment, the combination of the lowest concentration of MC extract (10 µg/L) with either concentration of *L. platensis* extract significantly increased the Zn content compared to the treatment with both concentrations of MC (10 and 40 µg/L). However, when the highest concentration of MC extract was applied in combination, the Zn content remained significantly lower (*p* < 0.001) than that of the control.

Taproots of radish plants treated with both concentrations of MCs (10 and 40 µg/L) as well as with *L. platensis* extracts (0.1 and 1 g/L) showed a significant reduction (*p* < 0.001) in calcium (Ca) content relative to the control. Similarly, co-treatment with MCs and *L. platensis* extracts also resulted in a significant decrease (*p* < 0.001) in Ca levels. By contrast, application of *L. platensis* extract alone (0.1 and 1 g/L) significantly (*p* < 0.001) enhanced phosphorus (*p*) content, whereas the highest concentration of MCs extract (40 µg/L) caused a significant reduction compared with control. Interestingly, co-treatment with all combinations of MC extract and *L. platensis* extract showed a significant increase (*p* < 0.001) in P content compared to the control and the treatment with MC alone.

## 4. Discussion

The findings of this study demonstrate that the presence of MCs in irrigation water, even at environmentally relevant concentrations (10 and 40 µg/L), exerts dose-dependent effects on the growth, oxidative stress markers, and nutritional quality of radish under hydroponic conditions. Morphologically, exposure to MCs led to a significant decline in leaf number, leaf area, plant height, and fresh biomass. These observations are in line with previous reports describing growth inhibition in plants exposed to MCs. For instance, Haida et al. [9] reported a reduction in fresh biomass, leaf number, and root length in *Fragaria vulgaris* exposed to 20 µg/L of MCs. Similarly, irrigation with water containing 1–20 µg/L of MCs led to a decrease in fresh weight in *Lactuca sativa* and *Lepidium sativum* [37,38], inhibition of root growth in *Medicago sativa* and *Lactuca sativa* [39], as well as reduced root elongation, crown root initiation, and lateral root development in *Brassica rapa* [37]. These results can be explained that under hydroponic conditions, MCs are likely more bioavailable, allowing direct contact with the root system, which may explain the strong effects observed even at low doses. The toxic action of MCs is mainly attributed to their strong inhibitory effect on serine/threonine protein phosphatases, key enzymes regulating essential processes such as metabolism, cell division, development, and gene transcription and translation in both animals and higher plants [40,41]. Supporting this, Saqrane et al. [42] demonstrated that the inhibition of these phosphatases in plants could lead to leaf malformations, histological alterations, and delays in root organ differentiation and vascular cylinder formation, accompanied by inhibition of lateral root primordia development.

In contrast, when *Raphanus sativus* L. was cultivated under the same hydroponic conditions but supplemented with an aqueous extract of *L. platensis* (0.1 and 1 g/L), a cyanobacterium that does not produce MCs, the plants displayed a significant improvement in several growth traits, including leaf number, leaf area, and fresh biomass of both roots and shoots (Table 3). These findings are consistent with previous research reporting that hydrolysates or extracts of *L. platensis*, applied at different concentrations (1–9 g/L), enhance plant growth and development. Such biostimulant activity has been reflected in increased plant biomass and height, greater leaf production, earlier flowering, higher floral biomass, more flowers, and increased stem number in several plant species, including *Lactuca sativa* [43], *Eruca sativa* [44], *Beta vulgaris* [45], *Amaranthus gangeticus,* and *Brassica rapa* [46], *Petunia x hybrida* [47], and *Solanum lycopersicum* [48]. The analysis of physiological traits revealed that the aqueous extract of *L. platensis* is particularly rich in total sugars, proteins, and auxins. These findings are consistent with previous reports highlighting the abundance of proteins, carbohydrates, macro- and micronutrients, polyamines, vitamins, enzymes, and hormone-like compounds in *L. platensis* [47,49]. Among these components, polysaccharides represent a major fraction of microalgal extracts and are known to play a key role in promoting plant growth [50]. Likewise, auxins, as central phytohormones, regulate fundamental processes such as cell division and elongation, tissue differentiation, apical dominance, abscission, and flowering [51].

The significantly higher growth rates, chlorophyll pigment levels, and soluble sugar contents recorded in radish plants cultured hydroponically with the application of *L. platensis* aqueous extract (0.1 and 1 g/L) (Figure 1 and Figure 2) raised an important question: can this extract confer protection against the phytotoxic effects induced by MCs? To address this, radish plants were exposed either to MCs alone (10 and 40 µg/L) or in combination with *L. platensis* extract (0.1 and 1 g/L), and their responses were assessed at the physiological, biochemical, and nutritional levels. Application of the aqueous extract at 1 g/L markedly alleviated the negative effects of MC exposure, leading to improvements in key growth parameters, including leaf number, leaf area, shoot and root length, and fresh biomass of both aerial and root tissues (Table 3). These findings align with earlier reports indicating that *L. platensis*-based biostimulants can mitigate the detrimental impact of various abiotic stresses on plants [52]. For instance, *L. platensis* suspension enhanced growth and productivity in salt-stressed *Vicia faba* L., an effect attributed to osmoregulatory metabolites such as proline, which are known to counteract reactive oxygen species (ROS) [53]. In the present study, growth inhibition observed after chronic exposure (45 days) to MCs is likely linked to disruptions in photosynthetic activity. This was reflected in reduced levels of photosynthetic pigments, notably chlorophylls and carotenoids, which in turn slowed plant development (Figure 1). Saqrane et al. [42], who found that irrigation with MC-contaminated water decreased chlorophyll a and b in the leaves of *Zea mays*, *Lens esculenta*, and *Triticum aestivum*, reported similar observations. Since these pigments are essential for light capture and energy transfer, their depletion directly hampers photosynthetic efficiency and plant performance [54]. Interestingly, supplementation with L. platensis extract (0.1 or 1 g/L), a non-MC-producing species, significantly enhanced chlorophyll b and total chlorophyll levels compared to the control, although no effect was recorded for carotenoids. These results corroborate previous findings where spirulina extracts boosted chlorophyll a and b in *Lupinus luteus* L. [55], and increased pigment levels when applied as foliar sprays on *Beta vulgaris* [39], or as supplements in sprouted radish leaves [44]. The stimulatory effect of spirulina is thought to be mediated by improved cell membrane permeability and nutrient uptake efficiency, particularly nitrogen, which is directly linked to chlorophyll biosynthesis [56]. Co-treatment of radish plants with MCs (10 and 40 µg/L) and *L. platensis* extract (1 g/L) resulted in a significant increase in chlorophyll b compared to MCs exposure alone, although levels remained lower than those obtained with *L. platensis* extract alone. This indicates a partial protective effect of the extract against MC-induced inhibition of chlorophyll b biosynthesis. However, even at the highest concentration (1 g/L), the extract failed to mitigate the MC-induced decline in carotenoid contents (Figure 1c).

At the biochemical level, exposure of radish plants to MCs (10 and 40 µg/L) significantly increased soluble sugar contents in both leaves and roots compared to the control. However, the increase was more pronounced in plants treated with *L. platensis* aqueous extract. Similar observations have been reported in *Beta vulgaris* and *Eruca sativa*, where foliar application of *L. platensis* extracts enhanced sugar accumulation [44,45]. In the present study, co-treatment with MCs and *L. platensis* extract (0.1 and 1 g/L) led to significantly higher sugar levels in both leaves and roots compared to MC exposure alone, though values remained lower than those obtained with *L. platensis* extract alone. Sugars are known to act as protective metabolites by stabilizing cellular membranes and functioning as antioxidants, thereby improving plant tolerance to stress [56]. Regarding proteins, both MC concentrations (10 and 40 µg/L) caused a significant reduction in leaf protein content relative to the control, while no effect was detected in roots (Figure 2). A similar decrease in protein levels was reported in *Lactuca sativa* leaves exposed to high concentrations of MC-LR (0.5–10.0 µg/L), MC-RR (0.15–3 µg/L), and total MCs (0.65–13.0 µg/L) [57]. In contrast, Haida et al. [9] observed reductions in protein content in both roots and leaves of *F. vulgaris* irrigated with MC-contaminated water (20 µg/L), whereas El Khalloufi et al. [8] reported increased protein levels in *Medicago sativa* exposed to 1–10 µg/L of MCs. These discrepancies likely reflect differences in the ability of MCs to inhibit protein synthesis while simultaneously stimulating the biosynthesis of antioxidant enzymes as a protective strategy against ROS [58]. In the current study, supplementation with *L. platensis* extract (0.1 and 1 g/L) effectively mitigated the MC-induced reduction in leaf protein content, restoring values to control levels. This protective effect may be attributed to the presence of vitamins and other bioactive compounds in spirulina extracts that promote protein and sugar synthesis [59].

In addition to inhibiting serine/threonine protein phosphatases [40], MCs can induce oxidative stress through mechanisms such as free radical generation, glutathione depletion, and lipid peroxidation, which represent another key pathway underlying their toxicity in both animal and plant cells [60]. Phenolic compounds are central to plant defense mechanisms against abiotic and biotic stresses [61]. Their accumulation is often regarded as an adaptive strategy, as they act as potent antioxidants involved in detoxifying free radicals, thereby maintaining essential plant functions under cyanotoxin-induced stress [62,63]. In the present study, treatment with MCs at both tested concentrations (10 and 40 µg/L) significantly increased polyphenol contents in radish leaves and roots compared to the control (Figure 4a,b). These findings align with previous studies reporting enhanced accumulation of phenolic compounds in various plant species exposed to MCs, including *Medicago sativa* (11.12 and 22.24 µg/mL) [22], *Fragaria vulgaris* (1–20 µg/L) [9], *Vicia faba* (50 and 100 µg/L) [64], and both *Raphanus sativus* and *Daucus carota* (1.5–33 µg/L) [65]. However, in this study we observed that treatment of the radish plant with the *Limnospira* extract at 1 g/L, the *L. plattensis* extract alone, and with the low concentration (10 µg/L) of MC extract and both concentrations of the *L. plattensis* extract induced a significant decrease in polyphenols content in leaves and root tissues, suggesting the protective potential against oxidative stress of this aqueous extract.

One of the primary events associated with oxidative stress in plants is the excessive production of ROS, which can overwhelm the antioxidant defense system and impair the plant’s ability to cope with contaminant-induced stress, ultimately leading to reduced biomass accumulation [66]. This defense system comprises non-enzymatic components (vitamins, polyphenols, carotenoids, glutathione) as well as enzymatic antioxidants such as catalase (CAT), superoxide dismutase (SOD), and peroxidase (POD). In the present study, in addition to the significant reduction in carotenoid levels in leaves (Figure 1) and the accumulation of polyphenols in leaves and roots (Figure 5), exposure of radish plants to environmentally relevant concentrations of MCs (10 and 40 µg/L) significantly altered the activities of SOD, POD, and CAT, enzymes that play a central role in maintaining redox balance by scavenging ROS. In leaf tissues, a significant increase in the activities of POD, SOD, and CAT was observed only at the higher MCs concentration (40 µg/L) compared to the control (Figure 6, Figure 7 and Figure 8). By contrast, in root tissues, all three enzymatic activities were significantly enhanced at both MC concentrations relative to the control. These findings are consistent with previous reports in other plant species. For instance, significant increases in POD and CAT activities were observed in the leaves and roots of *Medicago sativa* and *Vicia faba* exposed to 10–100 µg/L of MCs [8,64]. Similarly, elevated activities of CAT, SOD, and POD were reported in seedling cells of *Cucumis sativus* and *Oryza sativa* after 14 days of exposure to 100 µg/L of MCs [67]. Enhanced POD activity was also recorded in *Eruca sativa* after 15 days of exposure to 75–100 µg/L of MCs [68], while SOD and CAT activities were increased in *Lactuca sativa* and *Brassica rapa* subjected to higher MC concentrations (30–6400 µg/L) [69]. The upregulation of these antioxidant enzymes likely represents a crucial adaptive mechanism by which plants resist abiotic stress induced by MCs and regulate ROS homeostasis [69,70,71]. Together with polyphenols and carotenoids, the increased activities of POD, SOD, and CAT contribute to scavenging toxic free radicals and provide partial protection against lipid peroxidation during both acute and chronic MCs exposure.

Exposure of radish plants to both concentrations (0.1 and 1 g/L) of the aqueous extract of *L. platensis* significantly increased the activities of SOD, POD, and CAT in root tissues, while in leaf tissues only SOD and POD activities were enhanced (Figure 6, Figure 7 and Figure 8). Under MC-induced stress conditions, the aqueous extract of *L. platensis* further stimulated the activities of all three antioxidant enzymes (SOD, POD, and CAT) in both leaves and roots. This upregulation of enzymatic activities clearly indicates that the aqueous extract of *L. platensis* strengthens the enzymatic defense system of radish plants exposed to MCs, thereby improving their capacity to scavenge ROS. These findings are in agreement with previous studies showing that *L. platensis* can stimulate antioxidant enzymes such as CAT, SOD, and POD under various stress conditions, thereby attenuating oxidative damage through efficient detoxification of ROS [72,73,74,75], and mitigating salt stress in salt-tolerant tomato species [76]. Mechanistically, SOD catalyzes the conversion of superoxide anion radicals (O2−) into H_2_O_2_ and O_2_, while CAT decomposes H_2_O_2_ into non-toxic H_2_O and O_2_. Consequently, SOD and CAT are often induced simultaneously during oxidative stress to maintain redox homeostasis [77]. Therefore, this study highlighted the beneficial effect of the biostimulant extract based on *L. platensis* on strengthening and improving the antioxidant potential of radish plants. This can be explained by the fact that *L. platensis* naturally contains antioxidant elements such as polyphenols and carotenoids and also by its role in enhancing antioxidant activities, which helps plants to rapidly eliminate toxic ROS and thus reduce their damage to metabolic processes [78].

Lipid peroxidation is a well-known mechanism of cellular injury and is widely used as an indicator of oxidative stress in plant and animal tissues [79,80]. Malondialdehyde (MDA), a stable end-product of lipid peroxidation, is commonly used as a biomarker to assess oxidative damage under stress conditions [74]. In the present study, exposure of radish plants to MCs led to a significant increase in MDA levels in both leaves and roots after 45 days of treatment with environmentally relevant concentrations (10 and 40 µg/L). These findings are consistent with previous work reporting elevated MDA levels in *Medicago sativa* exposed to MCs at concentrations as low as 0.5 μg/L [61]. The observed rise in MDA content reflects the inability of the antioxidant defense system to fully counteract excessive ROS production, resulting in cellular oxidative damage [81]. Interestingly, when radish plants were co-treated with the aqueous extract of *L. platensis*, MDA levels significantly decreased, reaching values comparable to those of the control group. This demonstrates the protective potential of the extract in mitigating lipid peroxidation and maintaining membrane integrity under MC-induced stress. Similar protective effects were reported by Gharib et al. [82], who observed that *L. platensis* at 0.1 g/L significantly reduced lipid peroxidation and lowered MDA content in *Rosmarinus officinalis*.

Several studies have demonstrated that the presence of MCs in irrigation water not only affects plant growth, biochemical parameters, and oxidative stress markers but also compromises taproot yield and nutritional quality [83,84,85]. In the present study, exposure of radish plants to environmentally relevant concentrations of MCs (10 and 40 µg/L) led to a significant reduction in taproot biometric parameters and yield, as well as in their nutritional quality (Table 4 and Table 5). For instance, treatment with 40 µg/L of MCs caused a marked decrease in taproot dry weight (TDW) compared to the control (Table 3). Although co-treatment with 40 µg/L of MCs and the aqueous extract of *L. platensis* (0.1 or 1 g/L) partially restored TDW, the values remained significantly lower than those of the control. Moreover, vitamin C levels in taproots significantly decreased following exposure to both concentrations of MCs, while co-treatment with *L. platensis* extract restored these levels significantly. A similar protective effect of *L. platensis* was also observed for Zn and P contents in radish taproots (Table 5).

## 5. Conclusions

MCs can be bioaccumulated in both leaves and edible parts (taproots), with the highest concentration measured in taproots. Co-exposure of the plant with the *L. platensis* extract induced a slight but non-significant decrease in MCs’ bioaccumulation in both tissues. Bioaccumulation of MCs in both edible parts and leaves negatively affected taproot quality and yield, as well as plant biochemical and morphological characteristics by inducing oxidative stress marked by a significant increase in MDA level in both roots and leaves. However, treatment with 1 g/L of *L. platensis* extract improved the defense mechanisms of the radish plant by significantly reducing the MDA level in leaves and roots to the control value. Moreover, the *L. platensis* extract enhanced the nutritional quality of the plant that was affected by MCs by significantly increasing the levels of vitamin C and some minerals like zinc and phosphorus in the edible part (taproots). The promising results of the biostimulant and protective potential of the *L. platensis* extract against the negative effects of MCs would deserve to be further explored in field studies with different crops.

## Figures and Tables

**Table 1:** Biochemical properties of *M. aeruginosa* and *L. platensis* extracts. The values are denoted as mean ± standard error. Nd: not detected.

	*L. platensis* Extract	*M. aeruginosa* Extract
Total soluble sugar content (mg/g DW)	15.23 ± 0.92	25.62 ± 2.5
Total phenolic content (µg Gallic acid/g DW)	0.2 ± 0.01	Nd
Proteins content (mg/g DW)	39.67 ± 2.11	30.23 ± 1.21
Flavonoids content (µg Catechin/g DW)	0.3 ± 0.03	Nd
Auxins content (mg/g DW)	44.69 ± 2.10	Nd

**Table 2:** Concentrations of MCs (µg equiv. MC-LR/kg FW) detected in radish (*Raphanus sativus*) tissues (leaves and taproots) after 45 days of exposure to *L. platensis*, MC extracts, and their combinations. Values are mean ± standard error (*n* = 3). Different letters indicate significant differences at *p* < 0.05 (Anova, Tukey’s HSD test). Nd: not detected.

		0 µg/L	10 µg/L	40 µg/L	0.1 g/L	1 g/L	10 µg/L + 0.1 g/L	10 µg/L + 1 g/L	40 µg/L + 0.1 g/L	40 µg/L + 1 g/L
MCs in radish tissues (μg/kg FW)	Taproots	Nd ^a^	10.13 ± 1.45 ^b^	14.64 ± 1.50 ^b^	Nd ^a^	Nd ^a^	9.68 ± 2.84 ^b^	9.04 ± 0.73 ^b^	12.53 ± 1.99 ^b^	10.15 ± 0.38 ^b^
	Leaves	Nd ^a^	7.33 ± 0.14 ^b^	8.81 ± 1.35 ^b^	Nd ^a^	Nd ^a^	5.90 ± 0.65 ^b^	6.63 ± 0.10 ^b^	5.88 ± 0.60 ^b^	5.35 ± 0.25 ^b^

**Table 3:** Effects of *L. platensis* (LP), MC extracts, and their combinations at different concentrations on radish growth and yield parameters after 45 days of treatment. Values are mean ± standard error (*n* = 3). Different letters indicate significant differences at *p* < 0.05 (Anova, Tukey’s HSD test). LN: Leaves Number; LA: Leaf area (cm^2^); APL: Aerial part Length (cm); RL: Root Length (cm); RFW: Root Fresh Weight (g); LFW: Leaf fresh weight (g); TL: Taproot length (cm); TFW: Taproot fresh weight (g); TDW: Taproot dry weight (g); TD: Taproot diameter (cm).

	Growth Parameters (Leaves and Roots)						Yield Parameters (Taproots)			
	LN	LA (cm^2^)	APL (cm)	RL (cm)	LFW (g)	RFW (g)	TL (cm)	TFW (g)	TDW (g)	TD (cm)
Control	5 ± 0.57 ^a^	15.28 ± 0.62 ^ab^	12.33 ± 1.58 ^a^	16.03 ± 1.18 ^a^	1.75 ± 0.15 ^ab^	2.05 ± 0.08 ^a^	4.31 ± 0.27 ^a^	8.03 ± 0.67 ^ab^	2.37 ± 0.34 ^bcd^	4.22 ± 0.33 ^abc^
10 µg/L of MCs	5.66 ± 0.88 ^ab^	11.67 ± 0.12 ^ab^	12.6 ± 0.90 ^a^	14.63 ± 4.53 ^a^	1.5 ± 0.6 ^a^	2.50 ± 0.15 ^ab^	3.17 ± 0.17 ^a^	7.47 ± 0.23 ^a^	1.47 ± 0.23 ^abc^	3.71 ± 0.14 ^ab^
40 µg/L of MCs	5.66 ± 0.33 ^ab^	5.24 ± 0.88 ^a^	10.3 ± 1.24 ^a^	14.5 ± 1.51 ^a^	0.9 ± 0.1 ^a^	1.76 ± 0.40 ^a^	4.03 ± 0.58 ^a^	6.73 ± 0.20 ^a^	0.83 ± 0.13 ^a^	3.17 ± 0.03 ^a^
0.1 g/L of LP	8 ± 0.57 ^bc^	18.20 ± 0.10 ^ab^	10.43 ± 0.26 ^a^	16.8 ± 0.81 ^a^	1.9 ± 0.8 ^ab^	1.85 ± 0.14 ^a^	4.57 ± 0.28 ^a^	8.26 ± 0.07 ^abc^	2.26 ± 0.07 ^abc^	4.33 ± 0.24 ^abc^
1 g/L of LP	9 ± 1 ^c^	49.31 ± 0.64 ^c^	13.36 ± 1.07 ^a^	18.73 ± 2.49 ^a^	3.1 ± 0.5 ^cd^	12.33 ± 1.07 ^bc^	4.83 ± 0.50 ^a^	9.82 ± 0.20 ^c^	3.82 ± 0.20 ^d^	5.37 ± 0. 33 ^c^
10 µg/L of MCs + 0.1 g/L of LP	5.66 ± 0.33 ^ab^	26.15 ± 8.70 ^b^	12.7 ± 0.72 ^a^	14.46 ± 1.10 ^a^	1.35 ± 0.35 ^a^	2.3 ± 0.28 ^a^	3.75 ± 0.43 ^a^	7.9 ± 0.17 ^ab^	1.9 ± 0.17 ^abc^	4.11 ± 0.07 ^ab^
10 µg/L of MCs + 1 g/L of LP	7.66 ± 0.33 ^abc^	48.18 ± 2.48 ^c^	14.9 ± 1.08 ^a^	17.33 ± 1.47 ^a^	2.25 ± 0.35 ^abc^	3.83 ± 0.43 ^c^	4.07 ± 0.22 ^a^	8.53 ± 0.52 ^bc^	2.83 ± 0.71 ^cd^	4.46 ± 0.23 ^bc^
40 µg/L of MCs + 0.1 g/L of LP	7 ± 0.57 ^abc^	16.62 ± 1.45 ^ab^	11.3 ± 0.37 ^a^	16.63 ± 3.68 ^a^	2 ± 1 ^ab^	2.1 ± 0 ^a^	4.1 ± 0.52 ^a^	7.07 ± 0.18 ^ab^	1.07 ± 0.19 ^ab^	3.28 ± 0.12 ^ab^
40 µg/L of MCs + 1 g/L of LP	6.66 ± 0.33 ^abc^	47.99 ± 5.58 ^c^	15.2 ± 1.49 ^a^	16.73 ± 3.20 ^a^	3.55 ± 0.35 ^c^	3.75 ± 0.08 ^bc^	4.32 ± 0.49 ^a^	7.13 ± 0.12 ^ab^	1.18 ± 0.17 ^ab^	3.77 ± 0.42 ^ab^

**Table 4:** Biochemical characteristics of radish taproots after 45 days of exposure to different concentrations of *L. platensis* and MC extracts, applied individually or in combination. Values are mean ± standard error (*n* = 3). Different letters indicate significant differences at *p* < 0.05 (Anova, Tukey’s HSD test).

	Biochemical Parameters (Taproots)			
	Sugar (mg/100 g of FW)	Proteins (mg/100 g of FW)	Polyphenols (Gallic Acid mg/100 g of FW)	Vitamin C (Ascorbic Acid mg/100 g of FW)
Control	12.13 ± 1.13 ^a^	93.72 ± 0.74 ^abc^	306.45 ± 37.90 ^a^	24.94 ± 0.92 ^c^
10 µg/L of MCs	11.77 ± 0.10 ^a^	79.51 ± 6.78 ^ab^	323.45 ± 24.73 ^a^	15.85 ± 1.83 ^ab^
40 µg/L of MCs	11.10 ± 0.39 ^a^	75.07 ± 7.29 ^a^	354.49 ± 5.15 ^a^	12.68 ± 0.92 ^a^
0.1 g/L of LP	14.02 ± 0.82 ^a^	107.13 ± 4.68 ^c^	290.58 ± 37.47 ^a^	53.36 ± 1.91 ^de^
1 g/L of LP	15.54 ± 1.48 ^a^	112.25 ± 1.03 ^c^	277.44 ± 1.52 ^a^	69.21 ± 2.30 ^f^
10 µg/L of MCs + 0.1 g/L of LP	13.75 ± 2.58 ^a^	101.92 ± 8.03 ^bc^	292.64 ± 9.25 ^a^	45.97 ± 1.58 ^d^
10 µg/L of MCs + 1 g/L of LP	12.65 ± 2.01 ^a^	94.64 ± 0.75 ^abc^	298.73 ± 5.27 ^a^	54.95 ± 1.40 ^e^
40 µg/L of MCs + 0.1 g/L of LP	9.65 ± 0.92 ^a^	81.35 ± 3.78 ^ab^	300.25 ± 12.45 ^a^	28.53 ± 2.42 ^c^
40 µg/L of MCs + 1 g/L of LP	11.52 ± 1.20 ^a^	88.66 ± 7.88 ^abc^	310.89 ± 4.02 ^a^	21.56 ± 2.08 ^bc^

**Table 5:** Effects of different treatments on nutrient contents of radish taproots after 45 days of treatment with *L. platensis* and MC extracts and their combinations at various concentrations. Values are mean ± standard error (*n* = 3). Different letters indicate significant differences at *p* < 0.05 (Anova, Tukey’s HSD test). Fe: Iron; K: Potassium; Mg: Magnesium; Zn: Zinc; Ca: Calcium; P: Phosphorus.

	Nutrient Content (Taproots)					
	Fe (mg/100 g of DW)	K (mg/100 g of DW)	Mg (mg/100 g of DW)	Zn (mg/100 g of DW)	Ca (mg/100 g of DW)	P (mg/100 g of DW)
Control	0.207 ± 0.41 ^a^	4.68 ± 0.52 ^cd^	14.28 ± 0.35 ^c^	0.814 ± 0.083 ^c^	43.48 + 2.21 ^c^	165.22 + 11.48 ^bc^
10 µg/L of MCs	0.151 ± 0.01 ^a^	2.97 ± 0.15 ^bc^	13.72 ± 0.89 ^c^	0.509 ± 0.005 ^ab^	23.19 + 0.23 ^b^	137.36 + 24.21 ^ab^
40 µg/L of MCs	0.114 ± 0.004 ^a^	2.68 ± 0.07 ^ab^	1.78 ± 0.26 ^a^	0.352 ± 0.009 ^a^	14.03 + 0.29 ^a^	101.49 + 4.59 ^a^
0.1 g/L of LP	0.213 ± 0.05 ^a^	5.48 ± 0.29 ^d^	1.11 ± 0.21 ^a^	1.366 ± 0.005 ^d^	13.32 + 0.16 ^a^	290.56 + 11.48 ^ef^
1 g/L of LP	0.234 ± 0.01 ^a^	6.02 ± 0.11 ^d^	4.91 ± 4.11 ^ab^	1.678 ± 0.073 ^e^	11.95 + 0.07 ^a^	318.00 + 3.07 ^f^
10 µg/L of MCs + 0.1 g/L of LP	0.167 ± 0.005 ^a^	0.77 ± 0.06 ^a^	10.87 ± 0.39 ^bc^	0.707 ± 0.057 ^bc^	15.65 + 0.50 ^a^	251.07 + 2.52 ^de^
10 µg/L of MCs + 1 g/L of LP	0.163 ± 0.005 ^a^	1.96 ± 0.85 ^ab^	4.71 ± 0.66 ^ab^	0.815 ± 0.001 ^c^	15.30 + 1.02 ^a^	281.87 + 6.74 ^ef^
40 µg/L of MCs + 0.1 g/L of LP	0.162 ± 0.004 ^a^	0.89 ± 0.04 ^a^	6.96 ± 0.34 ^abc^	0.416 ± 0.005 ^a^	21.21 + 0.93 ^b^	197.48 + 13.25 ^cd^
40 µg/L of MCs + 1 g/L of LP	0.132 ± 0.001 ^a^	2.29 ± 0.17 ^ab^	1.53 ± 0.48 ^a^	0.53 4 ± 0.011 ^ab^	14.23 + 0.68 ^a^	220.76 + 3.82 ^d^

## Data Availability

The original contributions presented in this study are included in the article. Further inquiries can be directed to the corresponding author.
